# Association Between Sub-types of Sibling Bullying and Mental Health Distress Among Chinese Children and Adolescents

**DOI:** 10.3389/fpsyt.2020.00368

**Published:** 2020-05-14

**Authors:** Xiaoqun Liu, Chang Peng, Yizhen Yu, Mengsi Yang, Zaihua Qing, Xiaoyan Qiu, Xinhua Yang

**Affiliations:** ^1^Department of Maternal and Child Health, Xiangya School of Public Health, Central South University, Changsha, China; ^2^Department of Maternal, Child and Adolescent Health, School of Public Health, Tongji Medical College, Huazhong University of Science and Technology, Wuhan, China; ^3^Department of Student Affairs, Hunan University of Finance and Economics, Changsha, China; ^4^Institute of Higher Education, Hunan University of Science and Engineering, Yongzhou, China; ^5^Institute of Education, Hunan Agricultural University, Changsha, China

**Keywords:** sibling bullying, mental health, depression, anxiety, adolescents

## Abstract

Sibling bullying is a common phenomenon in childhood and adolescence worldwide and has a significant association with mental health distress. However, there have been few studies that have examined the associations between any specific sub-type of sibling bullying and depression as well as anxiety. Besides, the association between sibling bullying and psychological well-being was never explored among the Chinese population. The purpose of this cross-sectional study was to examine the associations between the number of sub-types of sibling bullying involvement and depression as well as anxiety among Chinese children and adolescents. Multi-stage stratified cluster sampling was used to recruit 5,926 participants aged 10 to 18 who had at least one sibling living in the household. Different sub-types of sibling bullying involvement were determined by using Olweus Bully/Victim Questionnaire (OBVQ). The nine-item Patient Health Questionnaire (PHQ-9) and the seven-item Generalized Anxiety Disorder Scale (GAD-7) were used to screen clinical ranges of major depression and generalized anxiety disorder, respectively. Of the participants, 1,235 (20.8%) were bullied by siblings, and 1,230 (20.8%) perpetrated bullying behavior against siblings over the past 6 months. After controlling potential confounders, adjusted model of logistic regression analyses indicated that all three sub-types of sibling victimization and perpetration were significantly associated with both depression and anxiety. There were linear associations between the number of sub-types of sibling bullying victimization and depression (adjusted OR = 1.49, 95% CI 1.32 to 1.68) as well as anxiety (adjusted OR = 1.68, 95% CI 1.48 to 1.90). Besides, linear trends were found between the number of sub-types of sibling bullying perpetration and depression (adjusted OR = 1.44, 95% CI 1.26 to 1.64) as well as anxiety (adjusted OR = 1.63, 95% CI 1.42 to 1.87). The findings underline dose–response relationships between the number of sub-types of sibling bullying involvement and mental health distress. Intervention programs should be conducted to focus on developing mental health status of those children and adolescents who are involved in multiple sub-types of sibling victimization or perpetration.

## Introduction

Sibling bullying refers to any unwanted, repeated, and harmful aggressive behavior among siblings (1), and it encompasses three major sub-types, including verbal (e.g., name-calling, teasing), physical (e.g., kicking, hitting), and relational (e.g., excluding from social situation, spreading rumors) bullying (2, 3). Compared to a substantial body of studies related to peer bullying, bullying behavior among siblings has received less research attention (4, 5). However, if all sub-types are considered, the prevalence of sibling bullying is much higher than that of peer bullying, which is perpetrated by a peer or a group of peers (6, 7). According to a systematic review including four quantitative studies conducted in different countries, nearly 50% of children are involved in sibling bullying every month, and 16% to 20% of them experience sibling bullying more than once a week (7–9). Though sibling bullying is usually considered by the parents as a normal or harmless phenomenon (10, 11), there is ample evidence that sibling bullying increases the odds of reporting a number of psychological and adjustment problems in childhood or early adulthood, which include depression (12, 13), anxiety (14, 15), emotional and conduct problems (9, 16), self-harm behavior (13), and even suicide (12). In addition, given the overlap of sibling and peer interactions in childhood and adolescence, previous studies have found that sibling bullying has significant association with peer bullying (3, 6).

Depression and anxiety are two of the most common psychiatric symptoms (17, 18). Many research studies have examined the associations between sibling bullying and depression or anxiety on the overall experiences of victimization (being bullied by siblings) or perpetration (perpetrating bullying behaviors to siblings) (13, 19). Yet, there were few studies to examine the associations based on any specific sub-type of sibling bullying involvement (15, 20). The previous studies suggest that associations between peer bullying and mental health problems are varied and complex since peer bullying involvement was categorized into different sub-types, including verbal, physical, and relational bullying (21, 22). In a cross-sectional study, Yen et al. found that only physical peer victimization was significantly associated with all dimensions of anxiety symptoms (17). Therefore, there is an important clinical implication to explore whether different sub-types of sibling bullying have distinct or similar association with depression as well as anxiety.

Existing literature has shown that there is a dose–effect relationship between the frequency of sibling bullying involvement and mental health distress. Indeed, those children and adolescents who were involved in sibling bullying victimization or perpetration several times a month or a week were at a particular risk of suffering psychological distress compared with those who never experienced sibling bullying or were involved in sibling bullying only ever once or twice (13, 19). Likewise, involvement in both sibling and peer victimization exhibited a dose–response association with psychological problems (6), with those were bullied both at home and at school having the highest odds for psychotic disorder (19). However, to the best of our knowledge, there is no study to explore the association between the number of sub-types of sibling bullying involvement and mental health problems. A previous study indicated that children and adolescents who were involved in multiple sub-types of sibling bullying victimization had reported greater mental health distress compared with those who experienced only one sub-type of sibling bullying victimization (15), and the effects seem to be cumulative (3). From this perspective, it is reasonable to explore if there is a dose–response association between the number of sub-types of sibling bullying involvement and depression or anxiety.

Aggressive behavior between siblings being a common form of family violence in childhood and adolescence worldwide and the fact that sibling bullying involvement can predict mental health problems (1, 23), in eastern countries, a small group of studies have analyzed the prevalence and risk of bullying, aggression, or violence betweem siblings (24, 25). Besides, there is no previous literature that has examined the link between sibling bullying and psychological well-being among a Chinese population (26). Although the one-child policy had been in effect in China since 1980, the implementation of the policy was less stringent in rural areas. People who lived in remote regions might be allowed to have more than one child when their first child was a girl or a disabled boy (27). On the other hand, the one-child policy was replaced by a universal two-child policy in 2015, and the number of families with two or more kids in both rural and urban areas is expected to increase reasonably (28). Therefore, it is necessary to explore the characteristics and potential risks of sibling bullying among Chinese population.

Taken together, there is a paucity of studies considering the association between different sub-types of sibling bullying involvement and mental health distress. Hence, we conducted a cross-sectional study with a large sample of Chinese children and adolescents to investigate (1) associations between three major sub-types (verbal, physical, and relational) of sibling bullying involvement (victimization and perpetration) and mental health distress (depression and anxiety) as well as (2) the associations between the number of sub-types of sibling bullying involvement and depression as well as anxiety. To this effect, we made some hypotheses: (1) all three major sub-types of sibling bullying have significant associations with mental health distress; and (2) there might be dose–response associations between the number of sub-types of sibling bullying involvement and depression as well as anxiety.

## Materials and Methods

### Procedures

This study was a cross-sectional survey conducted from April to July 2018. The participants were recruited from Hunan Province, China, by using multi-stage cluster sampling. We used a geography-based stratified sampling frame, which included three cities selected randomly from southern, central, and northern parts of the province, respectively. Three junior high schools and three senior high schools were selected randomly from each chosen city, and all the students of grade 7 to 12 were invited to the research. Before the field investigation, we requested permission from the principals of each school. Once the permissions were granted, investigators conducted the research in each class with the help of the form teachers. All participants signed informed consent forms, and the purpose of the study as well as the questionnaire sections were explained to them by investigators. The students were assured of the anonymity and confidentiality of the information provided in the self-reported questionnaires, and the respondents were free to discontinue their participation at any time of the study. The study received the approval from the ethical committee of Xiangya School of Public Health, Central South University.

The data collection was carried out by double input and through logical verification and sampling inspection of 10% of the input data to control the quality of data collection. If there was a problem with the input data, the researcher would check the original questionnaires in time to ensure the accuracy and reliability of the data.

### Participants

Questionnaires were sent out to 8,918 students from 18 sampled schools; of these, 8,520 (95.5%) completed the survey without apparent logical errors, and missing items were fewer than 15% of those in the questionnaire. Participants with no siblings (N = 2,415) and those aged 18 or above (N = 179) were excluded. The current study focused on 5,926 children and adolescents aged 10 to 18 who had at least one brother or sister living in the household at the time of the survey. Of the sample, 2,667 were boys (45.0%) and 3,259 were girls (55.0%). The average age was 14.55 ± 1.63. Most participants lived in a two-parent family (89.9%), while 566 children were from a single-parent family or other (9.6%). Of the 5,926 students, 4,518 had one sibling (76.2%), 1,114 had two siblings (18.8%), and 294 had three or more siblings (5.0%). Furthermore, 2,815 were the first child of their family (47.5%), 2,641 were the second-born child (44.6%), and 446 were the third-born or other birth order child in the household (7.5%).

### Assessment of Sibling and Peer Bullying

Sibling bullying was surveyed *via* the Chinese version of Olweus Bully/Victim Questionnaire (OBVQ). First, participants were provided with a definition of bullying according to Olweus that sibling bullying refers to any unwanted, repeated, and harmful aggressive behavior among siblings (29). Second, sibling bulling victimization was assessed by asking the participants whether they had ever been bullied by siblings in the last 6 months using the following six items: (1) having been hit, kicked, pushed, or shoved; (2) having belongings been taken or damaged; (3) having been called nasty name; (4) having been made fun of; (5) having been kept out of things on purpose, excluded from the group, or completely ignored; or (6) having had lies or rumors been spread about you and/or having had their sibling(s) try to make others dislike you. Then, sibling bullying perpetration was measured by asking whether the participant had even bullied their sibling(s) over the past half a year using the six items above. Items (1) and (2) relate to physical sibling bullying, (3) and (4) relate to verbal sibling bullying, and (5) and (6) relate to relational sibling bullying. The frequency was coded on a five-point scale ranging from 1 to 5 (1= never happened, 2 = only once or twice, 3 = two or three times a month, 4 = about once a week, and 5 = several times a week) (30). Children and adolescents were considered to be involved in any sub-type of sibling bullying victimization or perpetration if the frequency of bullying behavior mentioned above happened more than two or three times a month (2, 19).

For the status of sibling bullying, a pure victim was defined as his/her being involving in victimization but not engaging in perpetration, a pure bully was classified as his/her perpetrating bullying behavior but not being bullied, and a bully-victim was defined as his/her experiencing both victimization and perpetration of bullying. Those who neither bullied siblings nor were bullied by siblings were classified as “non-involved” (31).

Peer bullying was assessed by the Chinese version of OBVQ in the same way as sibling bullying, while the questions were adjusted for bullying behaviors between peers. Participants were asked whether they have had other students or friends bully them and had they ever bullied other students or their friends in the last 6 months using the six items as mentioned above. The Chinese version of Olweus Bully/Victim Questionnaire showed good reliability according to the existing literature (32).

In this study, the range of the internal consistency reliability (alpha Cronbach) for sub-scales of sibling bullying is from 0.76 to 0.83, and the Cronbach's alpha for sub-scales of peer bullying ranged from 0.77 to 0.84.

### Depression

The Chinese version of the nine-item Patient Health Questionnaire (PHQ-9) was used to screen depression of participants (33). The PHQ-9 has nine questions with a score ranging from 0 to 3 for each item and total score ranging from 0 to 27. A higher score indicates that the participant has more depressive symptoms. A total score of 10 or more is considered to meet the clinical range of major depression (1 = Yes, 0 = No) (34, 35). The prior study showed good reliability of the Chinese version of PHQ-9 in children and adolescent population (36). In the present study, the Cronbach's alpha of PHQ-9 was 0.88.

### Anxiety

The Chinese version of the seven-item Generalized Anxiety Disorder Scale (GAD-7) was used to assess anxiety disorders of children (37). The GAD-7 has seven questions with a score ranging from 0 to 3 for each item. Therefore, total score of GAD-7 ranges from 0 to 21 (38). A total score ≥ 10 of the GAD-7 is considered to meet the criterion for diagnosing generalized anxiety disorder (1 = Yes, 0 = No) (38, 39). The Chinese version of GAD-7 had good reliability among Chinese population according to early findings (40). In this study, the alpha Cronbach for GAD-7 was 0.93.

### Confounding Variables

Potential confounding factors were selected based on the literature with regard to the association between sibling bullying and psychological problems (12, 13), which included gender, age, number of siblings (one, two, or more than three), birth order (the first, the second, the third, or other), family composition (living in a two-parent family or a one-parent family), maternal education (primary school or less, junior high school, senior high school, college, or higher). Besides, parental conflict and parental abuse were considered as confounding variables in this study according to prior work (14, 25). Parental conflict and parental abuse were measured by asking participants “how often your parents fight with each other” and “how often your parents hit or abused you”. The frequency coded on a five-point scale in the last half year, ranging from 1 to 5 (1= never happened, 2 = only once or twice, 3 = two or three times a month, 4 = about once a week, and 5 = several times a week). Peer bullying (0 = not involved and 1 = involved in victimization or perpetration).

### Statistical Analysis

In our analyses, if the participant completed the survey with the missing items more than 15% in the questionnaire, we would exclude the whole information of the participant.

First, prevalence of participants involved in sibling bullying involvement was summarized by descriptive statistics [n (%)]. To assess associations between sibling bullying status (pure victim, pure bully, and bully-victim) and major depression as well as generalized anxiety disorder, two adjusted model of logistic regression analyses were performed separately. Second, for examining whether there were associations between different sub-types of sibling bullying and depression as well as anxiety, a set of adjusted model of logistic regression analyses were conducted to assess the associations after excluding those participants who were involved in any two or three sub-types of sibling bullying victimization or perpetration. Then, in order to examine the associations between the number of sub-types of sibling bullying involvement and depression or anxiety, a set of adjusted model of logistic regression analyses were performed with all participants who were categorized into four sub-groups (0 = participants not involved in any types of sibling bullying, 1 = participants involved in any one type of sibling bullying, 2 = participants involved in any two types of sibling bullying, and 3 = participants involved in all three types of sibling bullying). Finally, for testing if there is a dose–response relationship between the number of sub-types of sibling bullying involvement and depression or anxiety, we ran a set of adjusted model of logistic regression analyses again with the number treated as a continuous term.

Dependent variables of all adjusted logistic regression analyses mentioned above were major depression (total score ≥ 10) and generalized anxiety disorder (total score ≥ 10) separately. To address possible bias, potential confounding variables included gender, age, family composition, maternal education, number of siblings, birth order, parental conflict, parental abuse, and peer bullying. The associations were reported *via* adjusted odd ratios (OR) and 95% confidence intervals (95% CI). The significance level was set at *p* < 0.05. All of the statistical analyses were conducted by SPSS 22.0.

## Results

Of the 5,926 children and adolescents, 1,235 (20.8%) reported at least one sub-type of sibling bullying victimization over the past 6 months. The prevalence of three sub-types of sibling bullying victimization was 13.8% (verbal), 7.6% (physical), and 4.0% (relational). In addition, 1,230 (20.8%) participants had bullied their siblings with any sub-type of sibling bullying in the last half a year. The prevalence of three sub-types of sibling bullying perpetration were 14.5% (verbal), 6.5% (physical), and 2.8% (relational). With respect to sibling bullying status, 448 (7.6%) of children were pure victim, 443 (7.5%) were pure bully, and 787 (13.3%) were bully-victim.

In mental health distress, 1,171 (19.8%) and 875 (14.8%) children met a threshold score of diagnosing major depression and generalized anxiety disorder respectively (**Table 1**).

**Table 1:** The prevalence of sub-types of sibling bullying involvement, depression, and anxiety (N = 5,926).

	n (%)
Sub-types of sibling bullying victimization	
Verbal	818 (13.8)
Physical	450 (7.6)
Relational	237 (4.0)
Any	1235 (20.8)
Sub-types of sibling bullying perpetration	
Verbal	859 (14.5)
Physical	387 (6.5)
Relational	165 (2.8)
Any	1230 (20.8)
Depression	1171 (19.8)
Anxiety	875 (14.8)

### Associations Between Sibling Bullying Status and Depression and Anxiety

When looking at the status involved in sibling bullying (pure victim, pure bully, and bully-victim), results of adjusted logistic regression analyses indicated that any role of sibling bullying involvement was significantly associated with both depression and anxiety (**Table 2**).

**Table 2:** The associations between sibling bullying status and depression and anxiety [Adjusted OR (95% CI)].

	Sibling bullying status			
	Non-involved	Pure victim	Pure bully	Bully-victim
Bullying involvement (N = 5926)	4248	448	443	787
Depression (%yes)	16.8	28.3	25.5	27.4
Depression	1.00	1.73 (1.35-2.22)***	1.52 (1.17-1.98)**	1.71 (1.39-2.10)***
Anxiety (%yes)	11.8	21.7	20.1	23.6
Anxiety	1.00	1.85 (1.41-2.44)***	1.50 (1.12-2.01)**	2.09 (1.68-2.62)***

Significant confounding variables:

Depression = female gender, older age, living in a one-parent family, parental conflict, parental abuse, and peer bullying.

Anxiety = female gender, older age, living in a one-parent family, maternal education with college or more, parental conflict, parental abuse, and peer bullying.

**p < 0.01, ***p < 0.001.

### Associations Between Three Sub-types of Sibling Bullying Involvement and Depression and Anxiety

In victimization, after excluding 150 participants who experienced any two sub-types of sibling bullying victimization and 60 who were involved in all three sub-types of sibling bullying victimization, results of adjusted logistic regression analyses indicated that all verbal, physical, and relational victimization of sibling bullying were significantly associated with both depression and anxiety.

In terms of perpetration, after excluding 111 children or adolescents who acted any two sub-types of sibling bullying to their siblings and 35 who perpetrated all three sub-types of sibling bullying, there were significant associations between all three major sub-types of sibling bullying perpetration and depression as well as anxiety (**Table 3**).

**Table 3:** The associations between different sub-types of sibling bullying involvement and depression and anxiety [Adjusted OR (95% CI)].

	Sub-types of sibling bullying			
	Non-involved	Verbal only	Physical only	Relational only
Victimization (N = 5716)	4691	620	281	124
Depression (%yes)	17.7	22.6	29.5	29.8
Depression	1.00	1.38 (1.10-1.74)**	1.83 (1.35-2.47)***	1.71 (1.11-2.64)*
Anxiety (%yes)	12.6	18.1	21.7	24.2
Anxiety	1.00	1.65 (1.29-2.12)***	1.64 (1.16-2.30)**	1.93 (1.21-3.07)**
Perpetration (N = 5780)	4696	715	258	111
Depression (%yes)	17.9	23.9	30.6	26.1
Depression	1.00	1.46 (1.18-1.80)***	1.78 (1.30-2.44)***	1.72 (1.06-2.78)*
Anxiety (%yes)	12.8	18.7	24.4	23.4
Anxiety	1.00	1.55 (1.23-1.96)***	1.80 (1.27-2.54)**	1.87 (1.12-3.15)*

Significant confounding variables:

Depression (Victimization/Perpetration) = male gender, older age, living in a one-parent family, parental conflict, parental abuse, and peer bullying.

Anxiety (Victimization/Perpetration) = male gender, older age, living in a one-parent family, maternal education with college or more, parental conflict, parental abuse, and peer bullying.

*p < 0.05, **p < 0.01, ***p < 0.001.

### Associations Between the Number of Sub-types of Sibling Bullying Involvement and Depression and Anxiety

The adjusted model of logistic regression analyses indicated that children and adolescents had more risk of experiencing major depression when they reported being bullied by siblings with any one, two, or three sub-types of sibling bullying than those who were not involved in sibling bullying victimization. Meanwhile, there were significant associations between any number of sub-types of sibling bullying victimization and anxiety. What is more, a linear trend was found between the number of sub-types of sibling bullying victimization and depression (adjusted OR = 1.49, 95% CI 1.32 to 1.68, *p* < 0.001) as well as anxiety (adjusted OR = 1.68, 95% CI 1.48 to 1.90, *p* < 0.001).

The results of adjusted logistic regression analyses illustrated that participants who perpetrated any one, two, or three sub-types of sibling bullying would be more likely to report major depression than those who never acted bullying behavior to their siblings. Children and adolescents who acted any number of sub-types of sibling bullying had greater risk of anxiety compared to those who did not report sibling bullying perpetration. Afterwards, the number of sub-types of sibling bullying was treated as a continuous variable for logistic regression analyses. Liner associations were found between the number of sub-types of sibling bullying perpetration and depression (adjusted OR = 1.44, 95% CI 1.26 to 1.64, *p* < 0.001) as well as anxiety (adjusted OR = 1.63, 95% CI 1.42 to 1.87, *p* < 0.001) (**Table 4**).

**Table 4:** The associations between the number of sub-types of sibling bullying involvement and depression and anxiety [Adjusted OR (95% CI)].

	Number of sub-types of sibling bullying involvement				
	0	1	2	3	Linear trend
Victimization (N = 5926)	4691	1025	150	60	
Depression (%yes)	17.7	25.4	36.0	48.3	
Depression	1.00	1.54 (1.28-1.85)***	1.86 (1.26-2.76)**	3.77 (2.10-6.77)***	1.49 (1.32-1.68)***
Anxiety (%yes)	12.6	19.8	36.0	43.3	
Anxiety	1.00	1.65 (1.35-2.02)***	2.80 (1.88-4.17)***	4.90 (2.72-8.84)***	1.68 (1.48-1.90)***
Perpetration (N = 5926)	4696	1084	111	35	
Depression (%yes)	17.9	25.7	28.8	51.4	
Depression	1.00	1.56 (1.30-1.86)***	1.62 (1.03-2.55)*	2.89 (1.37-6.11)**	1.44 (1.26-1.64)***
Anxiety (%yes)	12.8	20.6	28.8	57.1	
Anxiety	1.00	1.61 (1.33-1.96)***	2.28 (1.44-3.61)***	5.79 (2.73-12.26)***	1.63 (1.42-1.87)***

Significant confounding variables:

Depression (Victimization/Perpetration) = male gender, older age, living in a one-parent family, parental conflict, parental abuse, and peer bullying.

Anxiety (Victimization/Perpetration) = male gender, older age, living in a one-parent family, maternal education with college or more, parental conflict, parental abuse, and peer bullying.

*p < 0.05, **p < 0.01, ***p < 0.001

## Discussion

This is the first study to examine the association between sibling bullying and mental health distress *via* a large and random sample in a non-western country. Moreover, the current study contributes new information with regard to the association from specific sub-type of sibling bullying involvement. The findings highlight the associations between three major sub-types of sibling bullying involvement and mental health problems. After controlling potential confounding variables, including individual characteristics, family characteristics, and other forms of family violence, we found that all verbal, physical, and relational victimization and perpetration of sibling bullying were associated with major depression as well as generalized anxiety disorder. More importantly, the number of sub-types of sibling bullying involvement is significantly associated with mental health distress in a dose–response fashion.

Confirmed our hypothesis, linear associations were found between the number of sub-types of sibling bullying involvement and depression and anxiety. Children and adolescents had higher odds of suffering major depression and generalized anxiety disorder with the number increasing, and those who were involved in all three sub-types of sibling bullying victimization or perpetration were at the highest risk of experiencing mental health distress. The finding extends existing studies that have identified dose–effect relationships between the frequency, the role, and the context of bullying involvement and mental health problems (13, 19). Therefore, target population of anti-bullying programs and mental health promotion must include not only those children who are involved in frequent bullying behavior (13), those who are bully-victims (a child that is both bully and victim) (2, 9), and those who are bullied by both siblings and peers (6) but also those who are involved in multiple types of victimization or perpetration.

Our work provides a new insight into the measurement of sibling bullying when examining the associations between bullying behavior among siblings and negative health outcomes. According to previous studies, sibling bullying involvement was generally treated as a binary term, and a victim or a bully of sibling bullying could be identified as long as any one item of the questionnaire met the cutoff value (13, 19). For example, a child would be classified as a victim when he or she had been hit by a brother or sister several times a month over the past half a year. Meanwhile, another child who had been hit, completely ignored, and made fun of by his or her siblings several times a month in the last 6 months was also identified as a victim of sibling bullying (19). From the findings of this study, the two children mentioned above may have different odds of experiencing mental health distress. Thus, future research should to treat sibling bullying involvement as a continuous variable according to the number of sub-types of sibling bullying in which participants are involved.

On the other hand, the cutoff value of sibling bullying in this study was “more than two or three times a month” (2, 19), which is different from some studies using the more stringent cutoff value, such as “more than once a week” (12, 31). When we chose “about once a week” as cutoff value in the study, the prevalence of sibling bullying was less than 5%, and it is much lower than 30.8% that reported in some western country (12). The difference might stem from different culture and concept of sibling relationship. Sibling bullying is a new issue but not a well-accepted topic in China in recent years. Chinese children and adolescents would avoid revealing their frequent aggressive behaviors among siblings because of traditional family values, which underline harmony and endurance.

Despite the potential contribution to the literature of sibling bullying, there are several limitations to this study. First, the nature of a cross-sectional survey limits our study to draw causality between sibling bullying and mental health distress. Moreover, there may be a bidirectional relationship between sibling bullying and depression or anxiety, which had been identified as the relationship between peer bullying and poor mental health outcomes (21, 41). Therefore, future longitudinal studies need to explore whether there is a bidirectional model between sibling bullying and mental health distress. Second, the study recruited children and adolescents aged 10 to 18 due to the fact that children aged under 10 may be unable to understand the questionnaire completely. However, the prevalence of sibling bullying peaks in childhood according to previous studies (6, 42). In the future study, we might invite younger children as our sample and information of investigation will be provided by their caregivers. In addition, the prevalence of sibling bullying in this study was measured by one of the siblings in a family through self-reported questionnaire, and there might be a information bias. Future research should consider the characteristics of sibling bullying behaviors and conduct research within a family and investigate bullying behaviors among all siblings. Finally, although the sample size of this study was rather large, the present study was conducted in a limited geographical setting. The extent to which this sample represents is unclear due to that the data of participants was only collected from students in Hunan Province, central China. Future research can recruit more children and adolescents in different regions of China.

Although there are limitations in some aspects of this study, our findings provide practical implications for clinicians, professionals, and policy makers. First, measure of sibling bullying should include specific sub-types of bullying behavior since they may have a cumulative effect on mental health outcomes. What is more, future research could take more sub-types of bullying into consideration, such as cyber bullying. At the same time, mental health education and promotion could be provided first to those children who are involved in multiple sub-types of sibling victimization or perpetration as they are at particular greater risk of mental health distress. In addition, since both sibling bullying and peer bullying are associated with depression as well as anxiety, effective preventive programs of anti-bullying should be conducted at home and at school simultaneously.

## Data Availability Statement

The raw data supporting the conclusions of this article will be made available by the authors, without undue reservation.

## Ethics Statement

The protocol was reviewed and approved by National Office for Philosophy and Social Science (Beijing, China). The study received the approval from the ethical committee of Xiangya School of Public Health, Central South University (grant number is XYGW-2017-056). Informed consents were obtained from the principal of each chosen school, the students who participated in the study, and their parents.

## Author Contributions

XL, as the first author, developed the initial manuscript. XL and MY were also responsible for the data collection and the data analysis. CP guided the overall design of the study. CP and YY also negotiated for program management of data and field access. ZQ, XQ, and XY contributed substantially to the revision and refinement of the final manuscript. All authors have read and approved the final manuscript.

## Funding

This work was supported by The National Social Science Fund of China (grant number is BBA170061).

## Conflict of Interest

The authors declare that the research was conducted in the absence of any commercial or financial relationships that could be construed as a potential conflict of interest.
